# Potential environmental factors influencing the occurrence of bottom shuffling in ordinary infants

**DOI:** 10.1111/ped.70304

**Published:** 2026-01-14

**Authors:** Ryoko Kalmar, Masakazu Umezawa

**Affiliations:** ^1^ Department of Early Childhood Education Mimasaka Junior College Tsuyama Japan; ^2^ Department of Materials Science and Technology, Faculty of Advanced Engineering Tokyo University of Science Tokyo Japan

**Keywords:** autonomy, bottom shuffling, environment, infant, motor development

## Abstract

**Background:**

Bottom shuffling (BS) occurs in ordinary infants without developmental disorders; however, the incidence and the related factors have been unclear. This study aimed to investigate the occurrence of BS in ordinary developing infants and the potential effects of parental intervention on the BS occurrence.

**Methods:**

A questionnaire survey on BS was conducted among 241 parents of 0‐year‐old and 1‐year‐old children in nursery schools, randomly selected from urban and rural areas in three prefectures in Japan.

**Results:**

Of the 241 infants without developmental disorders, 52 infants (21.6%) exhibited BS from 6 to 18 months of age (average: 8.3 ± 2.0 months). The ages of starting creeping, crawling, and unsupported sitting were 6.9 ± 1.8, 8.6 ± 1.8, and 7.0 ± 1.4 months of age, respectively. While the age of acquisition for creeping, crawling, or sitting did not differ due to BS, a correlation was found between BS incidence and parents' understanding of sitting development. Of the 241 parents, 110 parents responded that the timing of their infant's learning of “how to sit” should come just after their start of rolling over. In the infants with the 110 parents, a significant high frequency of the BS occurrence (27.3%, 30 out of 110) was observed.

**Conclusions:**

BS may lead to a decrease in opportunities for full‐body prone activity resulting in reduced physical activity, which raises concerns about the infant's autonomy.

## INTRODUCTION

A pediatrician usually assesses an infant's motor development during each medical consultation. Infants are expected to attain core skills related to motor development according to their age. A motor milestone has been a discrete event in a person's progressive mastery of control over the body's movements.^1^ Infant development is highly ordered and directional. According to Dr. Emmi Pikler, a 20th‐century Austro‐Hungarian pediatrician, infants normally develop the skills to turn over, sit up, stand up, and then walk in an intervention‐free environment where they are allowed to move their bodies independently.^2^ Recently, pediatricians and psychiatrists have considered the infants' developmental processes to be nonlinear and variable.^3^ with unordered higher‐order processes observed in daycares.^4^ Some infants learn to sit before turning over or walk without creeping or crawling first. Pediatricians assess developmental milestones at 18 months of age when infants typically begin to walk. If an infant acquires walking skills without creeping (do belly crawl) or crawling (hands‐and‐knees type with lifting one's belly off), recent studies showed that crawling experience affects motor skill development^5^, ^6^ and physical and cerebral development.^7^, ^8^, ^9^ In this study, we investigated bottom shuffling (BS) in ordinally infants, as a factor affecting crawling experience.

BS occurs before walking and involves moving their lower limbs while sitting. Downstream effects of BS include holding up without creeping or crawling and delays in starting to walk.^10^, ^11^, ^12^ Preterm births and some genetic conditions have been proposed to predispose infants to BS.^10^ BS increases sitting time before face‐down activity.^13^ BS has possible connections with autism and mental disabilities.^12^, ^14^, ^15^, ^16^, ^17^, ^18^

Preterm births and some genetic conditions have been proposed to predispose infants to BS.^10^ It has been observed that BS increases the time spent sitting before infants are active in a face‐down posture.^13^ Possible connections of BS with autism spectrum disorder and other mental disabilities have also been explored.^12^, ^14^, ^15^, ^16^, ^17^, ^18^ Research on BS focuses on diagnosis and early medical care. BS in children with normal development is called idiopathic shuffling,^19^ influenced by the environment and motor development.^20^ For example, passive sitting supported by adults can prevent natural play.^4^ Natural development involves turning over, raising their upper body with both hands from a face‐down posture, and searching by creeping or crawling on all four limbs.^2^, ^21^ Adults maintaining a passive sitting posture can cause BS, possibly because the infant's desire to search causes the infant to move from the sitting posture.^4^

It can, thus, be hypothesized that fixing and retaining the infant's sitting posture can cause BS, regardless of whether the infant's posture is superior to creeping or crawling on the floor, or the infant is in the process of acquiring skills to walk. In this study, we investigated the occurrence of BS in infants with ordinal development. We also examined the possibility that parental intervention affects the occurrence of BS.

## METHODS

### Preparation of questionnaire

**FIGURE 1:**
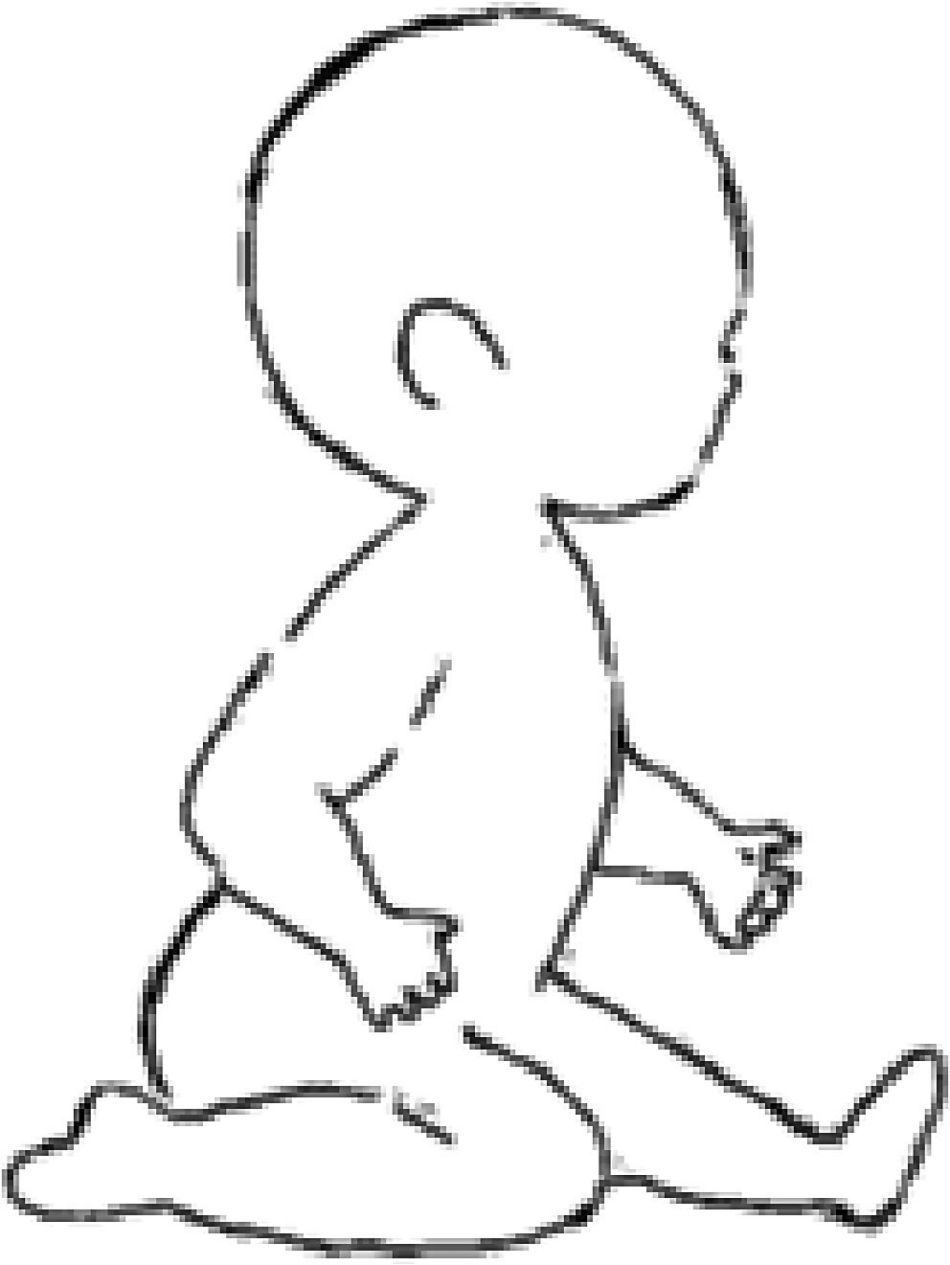
Illustration of shuffling.

The questionnaire prepared as described previously in detail contained the following questions^22^: [1] age and sex of the participants' children; [2] birth records including [2.1] gestation period, [2.2] birth weight, and [2.3] medical history; age at which the following motor development milestones were achieved: [3.1] being able to hold his/her head, [3.2] turning from supine to prone position, [3.3] sitting with support, [3.4] sitting without support (sitting‐up unsupported), [3.5] BS, [3.6] creeping, [3.7] crawling on both hands and knees, [3.8] holding on (standing up while grabbing), and [3.9] walking by themselves; [4] the participants' (parents') knowledge of when their child acquired sitting posture, based on the following five choices: (a) before turning from supine to prone, (b) at the same time as turning from supine to prone, (c) after turning from supine to prone, (d) after creeping and crawling, and (e) I do not know. In [3.5], BS is explained as follows: “Bottom shuffling is a method of movement where an infant sits and mainly uses their hips and bottom to move forward.” with an illustration (Figure 1).

### Participants

To investigate the environmental factors and lifestyle influences of different regions, we targeted three prefectures (Hyogo, Okayama, and Tottori) that include urban and rural areas. Furthermore, to make the research results more generalizable and to understand the impact on the behavior and development of infants from various backgrounds, we selected 31 nursery schools in both urban and rural areas based on the total population of each municipality. The target infants were enrolled in 0‐year‐old and 1‐year‐old classes, and those who had acquired walking were included. The target infants were 11–31 months old during the survey period as described previously.^22^ This target setting was based on a report by the Ministry of Health, Labour and Welfare that more than 90% of children developed walking by 16 months of age.^23^ The acquisition of walking after 18 months is considered a delay in walking during health checkups, and it is pointed out that this may be due to medical or developmental issues. These issues may include abnormalities in muscles or bones, problems with the nervous system, or developmental disorders. Therefore, such cases were excluded from the study. Personal response information was limited to numbers and was unlinked from names for analysis. This study was approved by the Ethics Committee of Hyogo University of Teacher Education (Approval No. 7, 2015). Written informed consent was obtained from all participants prior to their participation in the study.

**FIGURE 2:**
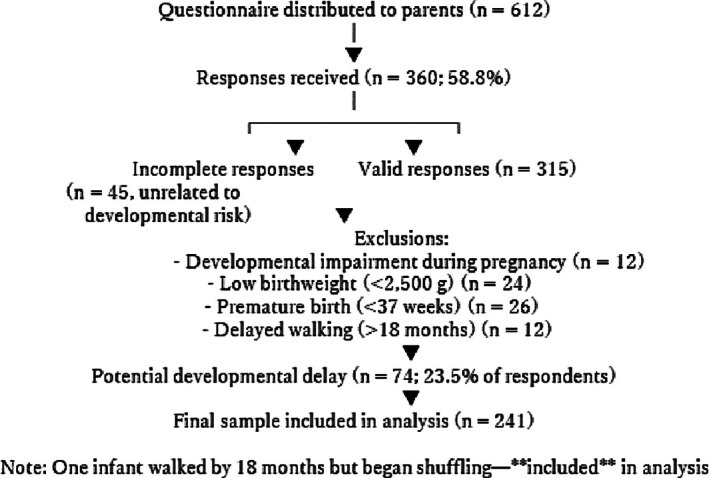
Flowchart of participant selection.

To facilitate the recruitment of participants, nursery school teachers were asked to distribute questionnaires to their parents. The request for responses and additional information was provided along with the survey form and questionnaire. The additional information stated that the responses were anonymous and voluntary, data would be statistically analyzed for research purposes, and personal information of the participants would be handled with care. The data were collected from their records in the governmental Maternal and Child Health Handbook, which are to be carried by parents from the child's gestation to school age, and must be presented at medical facilities, infant health checkups, and preschool entry. The coverage of the Handbook has been almost 100%^24^; therefore, reliable data could be collected. The questionnaire was distributed to a total of 612 parents, of whom 360 (58.8%) responded. Of these 360 cases, 61 (12 cases diagnosed with developmental impairment during pregnancy, 24 cases had low birthweight (<2500 g)), 26 cases with premature birth (born before 37 weeks of pregnancy). In infant health checkups, it is confirmed that infants have acquired independent walking by 18 months. Infants who have not acquired independent walking by 18 months are followed up due to concerns about developmental delays or low muscle tone. On the other hand, one infant had acquired walking by 18 months of age but began shuffling alongside walking as a means of movement at 18 months. This infant was included in the analysis. Therefore, we excluded 12 infants who started walking after 18 months from the analysis. In addition, 45 cases with incomplete responses were also excluded (Figure 2).

### Statistical analysis

The correlation between the months of acquisition of different milestones during the infants' gross motor development was analyzed using the Anderson‐Darling test. Then, the correlation between the starting age of BS and the starting age of creeping, crawling, and unsupported sitting was analyzed using Spearman's rank correlation test, a nonparametric test. To analyze the possible relationship between the occurrence of BS and the infants' parents' understanding of when they acquired a sitting posture, the 241 infants were divided into two groups (the non‐shuffling group or shuffling group) according to their parents' answers to the 4th question (a–e). The enrichment factor was defined to indicate the difference in the proportion of the shuffling infants in each category (a–e) as (nf/n)/(Nf/N), where nf is the number of shuffling infants in the category; n is the total number of infants within the same category; Nf is the total number of shuffling infants in any categories; and N is the total number of infants, 241. The normality of the data distribution was analyzed using the Anderson‐Darling test. The difference between the proportion of BS infants in each category was statistically analyzed using Fisher's exact test based on the hypergeometric distribution. After Bonferroni correction for multiple comparisons, the significance level was set at *p* < 0.05.

## RESULTS

**FIGURE 3:**
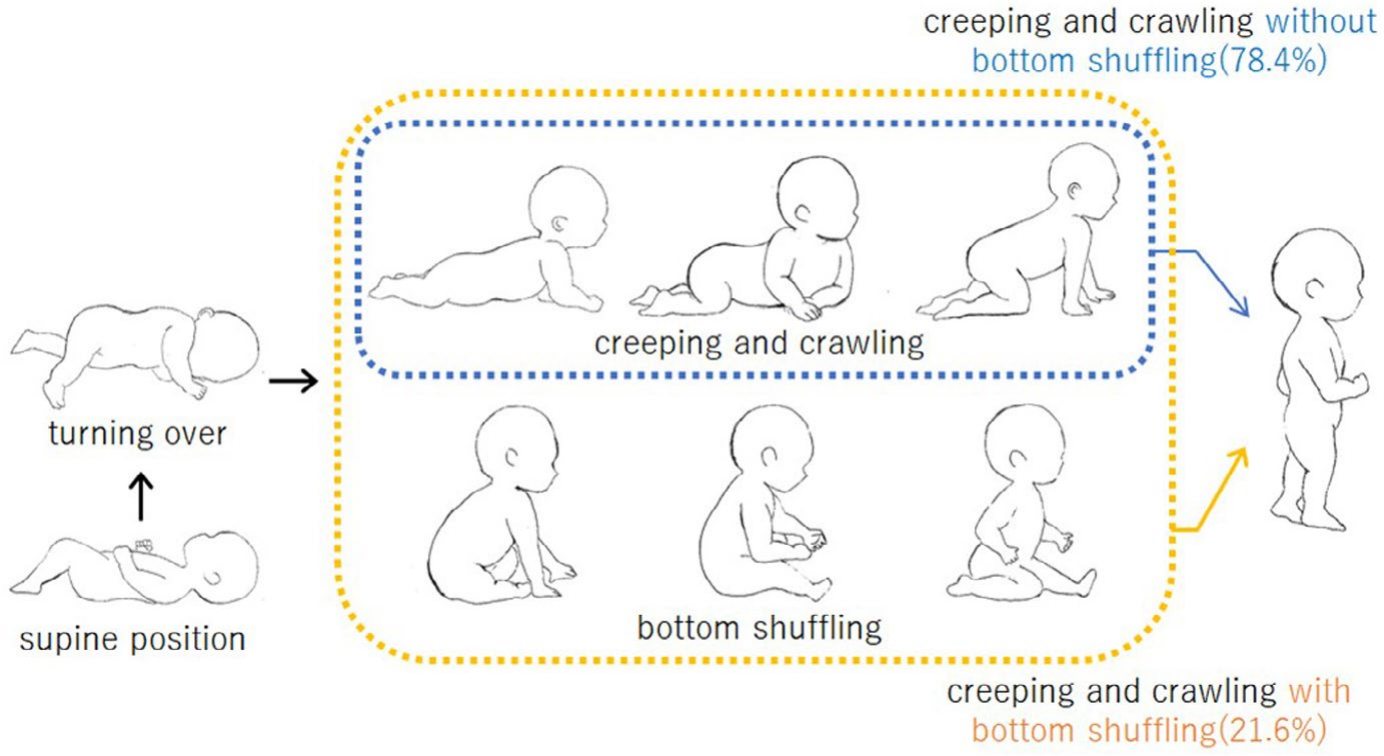
Developmental trajectories and prevalence of creeping, crawling, and bottom shuffling in infants.

**FIGURE 4:**
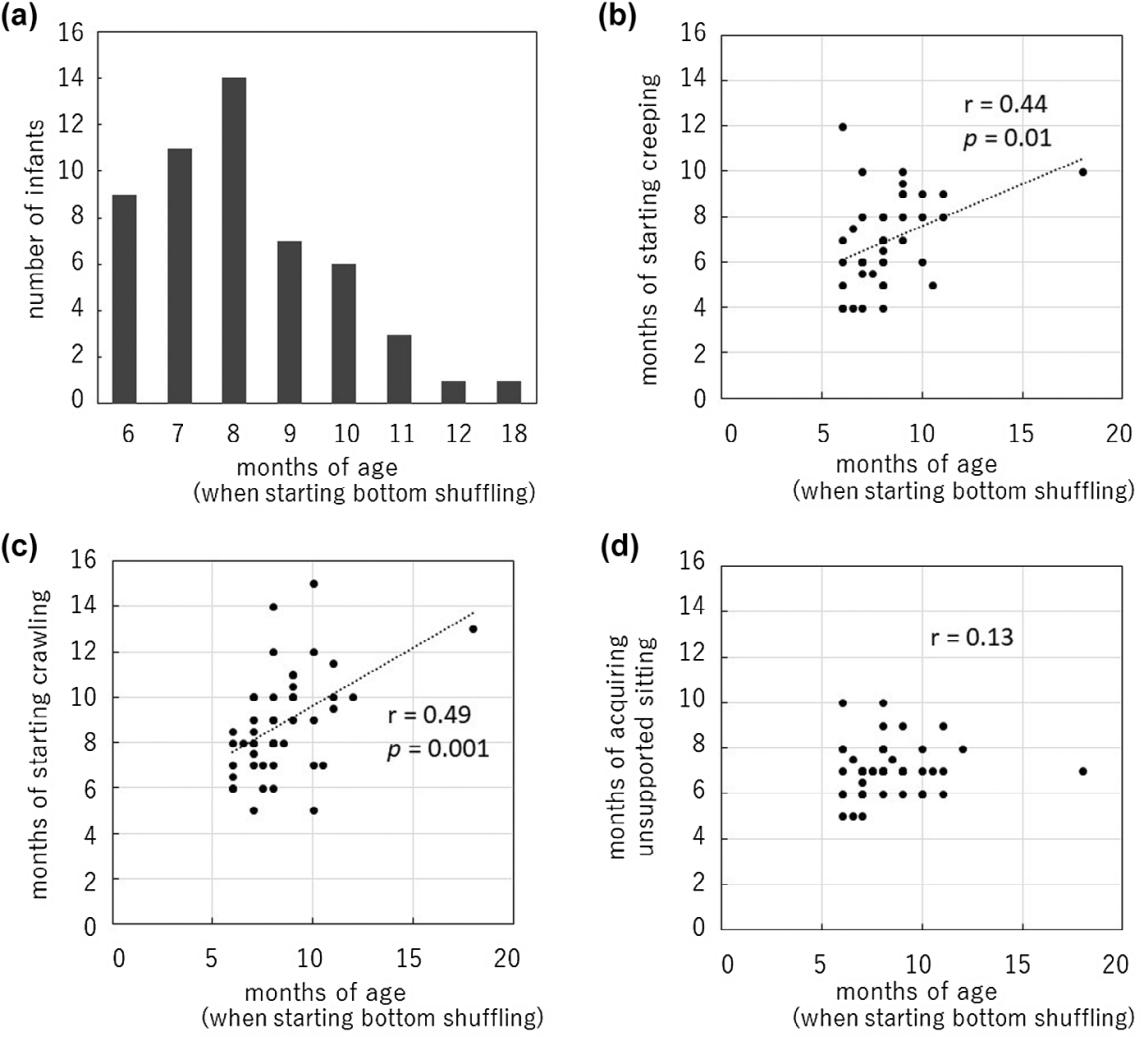
The occurrence of bottom‐shuffling and its relation to the other gross motor skills is acquired. (a) The month‐of‐age of occurrence of bottom‐shuffling (*N* = 52). (b–d) Scatter plots showing relationships between the age of starting bottom shuffling and the development of (b) creeping, (c) crawling, and (d) unsupported sitting.

In this study, we obtained responses from the parents of 241 infants without any developmental disorders and found that 52 out of 241 infants (21.6%) showed BS (Figure 3). The 52 infants started BS at 6–18 months of age (8.3 ± 2.0 months) (Figure 4a). The subjects were infants who had acquired independent walking by 18 months of age. However, infants who had experienced BS at 18 months old were considered to use both independent walking and BS as their means of mobility. Even after excluding this outlier, the correlation test demonstrated a similarly significant correlation.

The mean age for the acquisition of creeping, crawling, and unsupported sitting was 6.9 ± 1.8, 8.6 ± 1.8, and 7.0 ± 1.4 months, respectively. There was no difference in the age of acquisition of creeping, crawling, or sitting postures due to the occurrence of BS. We also investigated the relationship between the occurrence of BS and parental awareness of when infants acquired a sitting position. This awareness is not a parents' response which was affected by their infants' acquisition of sitting, but rather as a parents' perception about the timing of infants' sitting acquisition in general. BS occurred significantly more frequently in the category where parents responded that the infant developed the sitting position after turning over (30 out of 110 cases) (27.3%) than in the other categories (Table 1). The age at which BS started was significantly correlated with the age when creeping (*r* = 0.44, *p* = 0.01, Figure 4b) and crawling (*r* = 0.49, *p* = 0.001, Figure 4c) developed, while it was not correlated with that of unsupported sitting (*r* = 0.13, Figure 4d). When these 52 infants exhibiting BS were classified into early crawling and early sitting according to previous studies (24), 35 (67.3%) were early sitting. Of the infants who started BS at or before 8 months of age, 50.0% (17 of 34) were early sitting, whereas all the infants who started BS later than 8 months were early sitting.

**TABLE 1:** Relationship between the occurrence of bottom shuffling in infants and their parents' awareness of when their child developed the sitting posture.

Category	After creeping and crawling	After turning from supine to prone	At the same time as turning from supine to prone	Before turning from supine to prone	I do not know	Total
Number of shuffling infants	18	30	2	1	1	52
Number of non‐shuffling infants	81	80	14	2	12	189
Total	99	110	16	3	13	241
Percentage of shuffling infants within each category	18.2%	27.3%	12.5%	33.3%	7.7%	21.6%
Enrichment factor	0.84	1.26	0.58	1.54	0.36	
*p* value	0.07	0.02*	0.19	0.40	0.15	

*Note*: *p < 0.05.

## DISCUSSION

This study aimed to investigate the occurrence of BS in ordinal developing infants and to explore the potential influence of parental understanding and intervention on its development. Our findings revealed that 21.6% (52 out of 241) of infants exhibited BS, with its onset ranging from 6 to 18 months of age (mean 8.3 ± 2.0 months). All infants acquired independent walking by 18 months, though some continued to use both BS and walking as modes of mobility. Importantly, the ages of creeping, crawling, and unsupported sitting did not differ significantly between infants with and without BS. Interestingly, BS occurred significantly more often among infants whose parents believed that sitting was acquired after turning over (27.3%). Moreover, the onset of BS was significantly correlated with the ages of creeping (*r* = 0.40, *p* = 0.01) and crawling (*r* = 0.48, *p* = 0.001), but not with unsupported sitting (*r* = 0.08), indicating that crawling‐related movements may play a more prominent role in the emergence of BS than sitting alone.

Among infants who exhibited BS, 67.3% were classified as early in achieving the sitting position. Notably, all infants who began BS after 8 months were early sitters, while 50% of those who began BS at or before 8 months fell into this category. These findings suggest that infants who start BS at a later stage may have acquired a sitting posture before developing crawling skills, potentially reflecting a pattern of reduced crawling activity and increased reliance on supported sitting.

Previous research suggests that early sitters may exhibit decreased exploratory activity associated with crawling.^25^ In line with this, our results show that infants from the “early sitting” group started BS later, after 8 months of age. This supports the idea that the developmental sequence—particularly whether sitting occurs before crawling—may shape the infant's preferred modes of mobility.^22^ During the transition from crawling to standing, some infants may naturally progress through milestones, while others may rely more on adult assistance, such as being placed in a sitting posture before they are able to achieve it independently. This supported sitting may reduce the opportunity for crawling and increase the likelihood of BS.

Additionally, this study highlights the significant role of parental understanding and beliefs regarding motor development. Our findings suggest that parents' perceptions of the sequence of motor milestones may influence how they position or support their infants, which in turn may affect the infant's spontaneous movement patterns. As shown in cross‐cultural comparisons (e.g., Jamaican vs. British mothers),^26^ cultural context can further influence postural development, such as the timing and nature of sitting acquisition.

Taken together, these findings indicate that the posture in which infants are placed by caregivers—particularly with regard to supported sitting—may be a key factor influencing the emergence of BS. Respecting infants' autonomy and allowing self‐initiated motor exploration appear to be important for promoting natural developmental trajectories.

In Japan, regular infant health checkups are mandated by law, with the first official developmental screening conducted at the 18‐month checkup. However the number and frequency during the first year of life may still be insufficient to capture subtle variations in motor development such as BS. Considering the influence of parental perception and caregiving behavior on infants' motor trajectories, increasing the frequency or enhancing the content of developmental assessments during the 0‐year‐old period could serve as a public health priority. Early identification and guidance regarding posture and movement patterns, including BS, may help promote more balanced motor development and inform caregivers about appropriate support strategies.

Future research should further explore the relationship between early sitting, crawling suppression, and BS in different cultural and caregiving contexts, while also considering system‐level interventions such as optimizing the structure of early childhood health checkups.

This study is based on a questionnaire completed by parents and recorded in the Maternal and Child Health Handbooks. Given the rapid growth and developmental changes during infancy, it is undeniable that some records may become unclear or incomplete over time, which may affect the accuracy of reported motor milestones. This reliance on retrospective parental reporting constitutes a methodological limitation. Moving forward, we aim to conduct detailed longitudinal observations from early infancy to more precisely capture motor developmental trajectories. Through multidisciplinary collaboration—including pediatric, developmental, and public health perspectives—we hope to identify early risk factors, clarify causal relationships, and develop effective support strategies tailored to both cultural and caregiving contexts.

## AUTHOR CONTRIBUTIONS

R.K. is the project leader and conceived the overall research idea. R.K. performed experiments and collected data. M.U. substantially contributed to the data analysis. R.K. and U.M. wrote and edited the manuscript. All authors read and approved the final manuscript.

## FUNDING INFORMATION

This study was supported by a Grant‐in‐Aid for Scientific Research (KAKENHI) from the Japan Society for the Promotion of Science (JSPS), Grant Number: 25K06068 and by a staff research grant provided by Mimasaka University.

## CONFLICT OF INTEREST STATEMENT

This study was supported by a staff research grant from Mimasaka University and by JSPS KAKENHI Grant Number 25 K06068. The authors declare no other conflicts of interest related to this study.
